# Telomeric Ends and Telomerase—Canonical and Non-Canonical Roles in Breast Cancer Tumorigenesis and Therapy Resistance

**DOI:** 10.3390/biomedicines14020314

**Published:** 2026-01-30

**Authors:** Magdalena Kostrzewa, Julia Niedzielska, Wiktoria Mieczkowska, Maja Hoffmann, Julia Rypińska, Adrianna Kowalczyk, Magdalena Stachowiak, Błażej Rubiś

**Affiliations:** 1 The Student Scientific Society, Poznan University of Medical Sciences, Rokietnicka Str. 5, 60-806 Poznan, Poland; 87114@student.ump.edu.pl (M.K.); 88315@student.ump.edu.pl (J.N.); 87108@student.ump.edu.pl (W.M.); 87119@student.ump.edu.pl (M.H.); 87931@student.ump.edu.pl (J.R.); 87936@student.ump.edu.pl (A.K.); 2 Department of Clinical Chemistry and Molecular Diagnostics, Poznan University of Medical Sciences, Rokietnicka Str. 3, 60-806 Poznan, Poland; magdalena.stachowiak@student.ump.edu.pl; 3 Doctoral School, Poznan University of Medical Sciences, 60-812 Poznan, Poland

**Keywords:** telomerase, telomeres, breast cancer, cancer therapy, therapy resistance

## Abstract

Telomerase is known as a very specific marker of embryonic cells. It is responsible for telomere elongation (bypassing the end-replication problem) and thus supports normal cell division during tissue and organ development. But it is generally absent or very low in most normal adult somatic cells. However, its overexpression in adulthood (due to secondary expression and activity restoration) is commonly known to be associated with cancer. Apart from its canonical function (associated with telomere length restoration), it also carries out various other roles. Its non-canonical activity covers mitochondrial and epigenetic processes. Consequently, it contributes to the cell response to stress and chemotherapeutic drug treatment. A more detailed understanding of these phenomena offers the opportunity to identify new pathways and targets that may serve as critical factors in breast cancer diagnostics and therapy. In this article, we summarize the latest reports on the discovery of telomerase’s nature, including its canonical and non-canonical roles. The manuscript highlights how these mechanisms contribute to tumorigenesis, therapy resistance, and the survival of cancer cells. Understanding these multifaceted mechanisms behind hTERT’s role in (breast) cancer progression and therapy resistance is crucial for developing more effective therapeutic strategies.

## 1. Introduction

According to data from the International Agency for Research on Cancer in 2022, breast cancer is the second most prevalent cancer worldwide and the fourth leading cause of death among oncology patients. Countries with a high human development index (HDI; including Western European countries, such as Poland) show a higher incidence of this type of cancer, which is the most commonly diagnosed cancer among Polish women [1,2]. In contrast, countries with a low HDI, where access to effective diagnostics and treatment is limited, show a higher mortality rate among patients despite a lower incidence of breast cancer [1].

Cancer cells have developed mechanisms providing them the ability to successfully overcome the host defense systems, including resistance to proapoptotic signaling, secretion of growth factors, and suppression of anti-growth agents. In addition, tumor cells are insensitive to the immune system and are able to modify the surrounding environment by inducing angiogenesis and metastasis [3,4]. They are also characterized by unlimited proliferative potential, which is influenced by several factors, including the ability of cancer cells to restore telomeres after the incomplete replication that accompanies every somatic cell division. Consequently, the induced activity of this enzyme in oncological diseases determines the unlimited proliferative potential of cancer cells [5,6].

The above-mentioned function of telomerase, referred to as its canonical role, is not the only function of this enzyme in the cell. Non-canonical roles of this ribonucleoprotein complex include epigenetic and mitochondrial processes. Noteworthy, some of them are dependent on the localization of the key catalytic telomerase subunit that can be translocated from the cytoplasm to the mitochondria and vice versa. On the other hand, telomeric ends can form spatial forms, such as G-quadruplexes or i-motives, that can contribute to the DNA topology and thus affect processes related to therapy resistance [7,8,9,10]. All of the roles of telomerase and telomeres are far beyond just telomere restoration; they are critical for all human cells, including cancer cells. In this review, we summarize the balancing of telomerase on the edge of canonical and non-canonical activities in breast cancer, emphasizing its critical role in tumorigenesis, therapy resistance, and cellular homeostasis.

## 2. The Canonical Role of Telomerase in Breast Cancer

Telomerase activity is found in human cells during embryo development, but then it is significantly diminished (often up to undetectable levels) in most cells, with the exception of a couple of cells, including activated lymphocytes, germ cells, and some stem cells. But most normal somatic cells lack telomerase activity. In contrast, the enzymatic activity of telomerase is restored in most cancer cells (up to 90% of different cancer cells express the catalytic subunit of telomerase, hTERT, and show enzyme activity), which provides unlimited cell division potential that makes them immortal (they can overcome the Hayflick limit). On the other hand, this almost unique and characteristic cancer-cell feature makes telomerase an interesting and promising marker/target in therapeutic strategy [11].

Data assessment performed by Murillo-Ortiz et al. in 21 breast cancer patients showed that in 11 hTERT-positive cases, the telomerase catalytic subunit positively correlated with Her2-Neu [12]. Additionally, a significant telomere attrition after chemotherapy treatment was observed in these cases compared to hTERT-negative cases [12]. Another work by the same authors showed that the absence of ER-beta expression was associated with low levels of telomerase activity [13], suggesting the contribution of estrogen receptors in controlling telomerase expression and activity, which implies their contribution to the response to stress. In hormonally regulated tissues, telomere attrition, telomerase activity, and *hTERT* expression are under the control of steroid sex hormones and growth factors. Similarly, these factors are known to play a role in carcinogenesis via the regulation of *hTERT* levels or telomerase activity that reflects the response of cancer to therapy [14].

The telomerase complex is supported by the telosome or Shelterin. This complex consists of six proteins—TRF1, TRF2, POT1, RAP1, TIN2, and TPP1—and is responsible for stabilizing the T-loop telomeric structure, preventing chromosome end-to-end fusions and DNA degradation [15,16]. As reported by Motevalli et al., five out of six Shelterin proteins were significantly suppressed in several breast cancer cell lines, which could be associated with abnormal DNA methylation. It was demonstrated that DNA demethylation (5-aza-2′-deoxycytidine (5-aza-CdR) and trichostatin A (TSA)) in 21NT cells resulted in the increased expression of Shelterin proteins, followed by a substantial increase in telomere length [16]. This, in turn, leads to telomere stabilization that can trigger two alternative pathways. One of them limits the amount of genetic damage and clonal evolution of the cancer cell population. These tumors are considered more susceptible to treatment. Alternatively, telomere lengthening in cancer cells may contribute to chemoresistance. This might explain the fact that after chemotherapy, telomere length may significantly decrease, but it may also increase, leading to proliferative stress after treatment [17]. Thus, further detailed studies on the therapeutic potential of telomere- and telomerase-targeting are required.

### 2.1. Beneficial and Adverse Modulators of Telomerase Activity in Breast Cancer

The hope of reducing the unlimited proliferative potential of cancer cells and thus cancer regression is offered by telomerase inhibitors [11]. Those compounds are capable of targeting telomerase activity directly or repressing catalytic subunit expression. One of these compounds, Imetelstat (GRN163L), has been studied in clinical trials showing significant efficacy [18,19,20]. This oligonucleotide acts as a competitive antagonist by specifically binding to the TERC template region, thus preventing its association with telomeric DNA and causing complete telomerase inhibition. Its efficacy in breast cancer has been confirmed, but unfortunately, due to its significant cytotoxic effect, it cannot be used as a stand-alone therapy [20]. This is why its therapeutic potential has been studied in combination with other drugs, such as Herceptin (the FDA-approved therapeutic monoclonal antibody against HER2) and paclitaxel (works by binding to tubulin protein, stabilizing microtubules, preventing them from breaking down, which halts mitosis at the G2/M phase, leading to cancer cell death/apoptosis with or without bevacizumab, an anti-angiogenic agent) [18,21].

Another compound belonging to the oligonucleotides with anticancer potential is T-oligo, which is homologous to the 3′ telomeric overhang. They interact with telomeres by disrupting the structure of Shelterin. In addition, T-oligos exhibit anticancer properties by promoting the activation of the ataxia-telangiectasia-mutated pathway (ATM) and inducing cytotoxic effects by activating the cellular DNA damage response (DDR) [20]. The effectiveness of these oligonucleotides in breast cancer in vitro has already been confirmed [22], but bringing them to the clinical trial stage is difficult due to their inaccurately understood anti-tumor effect and rapid degradation by nucleases [20]. Nevertheless, their use in breast cancer cells treated with radiation therapy increases the chances of successful treatment [22].

Tara et al. conducted a study investigating an association between inhibition of the Aurora B kinase, using a compound called AZD1152-HQPA, and expression of telomerase in breast cancer cells. In comparison with normal breast tissue, malignant tumors exhibited significantly higher expression levels of Aurora B, which were particularly elevated in HER2-positive cancer. After treating SK-BR-3 and MCF-7 cell lines with AZD1152-HQPA for 48 h, the proliferation, long-term survival, and colony-forming capacity in both cultures were notably reduced. Real-time quantitative PCR revealed shortened telomeres. Additionally, Western blot confirmed a significant decrease in the expression of the hTERT protein. Researchers suspect MYC to be the mediator between Aurora B and hTERT, as it is the only *hTERT*-regulating transcription factor interacting with Aurora B, according to the STRING database [23]. With its promising nature, the efficacy and safety profile of Barasertib (AZD1152) has been further investigated in Phase I and I/II clinical trials of patients with other cancer types, such as acute myeloid leukemia and various solid malignant tumors [24,25,26]; however, clinical trials have yet to be conducted specifically concerning breast cancer patients.

In a different study, researchers found that insulin-like growth factor binding protein-3 (IGFBP-3) inhibits telomerase activity in breast cancer cells, as confirmed by telomere repeat amplification protocol (TRAP) and real-time PCR. This effect was attributed to a dose-dependent reduction in the levels of both the RNA component (TERC) and the catalytic subunit with telomerase reverse transcriptase (hTERT) activity. However, it should be noted that research on the effects of IGFBP-3 on telomerase activity in breast cancer is described as part of a complex signaling network, which requires further investigation [27]. Plant-derived substances represent a further widely researched group of compounds that could find use in breast cancer therapy. Boldine, a substance isolated from *Peumus boldus*, has shown the ability to downregulate *hTERT* expression in cells of the MCF-7 cancer line [28]. Barkhordari et al. examined the effects of another plant extract, helenalin, on breast cancer cells from the T47D line. They showed that cell proliferation decreased over time with increasing concentrations of the tested compound. Furthermore, real-time PCR verified the compound’s association with hTERT inhibition. However, the authors emphasize that further studies, particularly in vivo investigations, are required before helenalin can be considered a candidate for clinical research in breast cancer [29]. Moreover, the effects of curcumin found in turmeric on MCF-7 cells have been investigated, revealing multiple actions on breast cancer cells. In addition to promoting the production of necroptosis-related proinflammatory cytokines, such as IL-8 and TNF-α, it also inhibited the expression of telomerase subunit coding genes [30]. It is worth mentioning that not all compounds tested in studies on modifying telomerase activity in breast cancer contribute to a decrease in telomerase activity. Bisphenol A (BPA) and its analogs, bisphenol F (BPF) and bisphenol S (BPS), have been tested for their effects on telomerase. The trial was conducted on two breast cancer cell lines: the estrogen receptor (ER)-positive MCF-7 and the ER-negative MDA-MB-231. After 24 h of exposure to BPA, BPF, and BPS, a 2-3-fold increase in telomerase activity and expression was observed in MCF-7 cells, which was blocked by an estrogen receptor inhibitor (ERI). This phenomenon did not occur in MDA-MB-231 cells, suggesting that the effect of bisphenols depends on the presence of the estrogen receptor. After 48 h of treatment, a slight but ER-dependent telomere elongation was also observed in MCF-7 cells [31]. A brief overview of the research models in studies on modulators of telomerase activity in breast cancer is presented in Table 1. It is worth noting that an efficient and safe therapeutic approach requires studies to be conducted in reference, i.e., alongside normal/non-cancerous cells. Telomeres of the normal cells are longer than those in most cancer cells, and the level of telomerase activity is generally lower in normal telomerase-positive cells as compared with cancer cells. Thus, the risks associated with possible telomere shortening in normal cells because of off-target telomere shortening are perceived as relatively minimal. Therefore, the efficacy of telomerase inhibition leading to the loss of viability or apoptosis of cancer cells, combined with the relatively low risks of side effects in normal cells, indicates telomerase research as one of the most promising anticancer approaches [32].

### 2.2. G-Quadruplexes and i-Motif Structures

G4 formation can contribute to genome instability by inducing mutations, deletions, and promoting recombination events. Studies have shown the significance of G4 structures in malignant cells, particularly in breast cancer, suggesting their potential for cancer stratification and personalized treatment options. Due to their stability and presence in oncogenic promoters and telomeres, G4 structures are being explored as therapeutic targets to regulate the transcription of oncogenes containing G4 motifs in their promoter regions and to prevent telomere elongation in cancer cells [33]. The inhibition of telomerase activity through the stabilization of G4 structures and the activation of the DNA damage response via telomere uncapping can lead to cell proliferation arrest. Therefore, small molecules that target and stabilize G4 structures in the human genome could serve as promising and interesting cancer therapeutic agents [34].

G4 binders exhibit anticancer activities, including cell cycle arrest, DNA damage, senescence, and apoptosis. Recent studies demonstrated the anticancer effects of two G4-targeting ligands, BRACO-19 (B19) and C066-3108 (C066), which showed good selectivity toward telomeric G4 motifs, in prostate and breast cancer cell lines. In the aggressive triple-negative MDA-MB-231 breast cancer line, high DNA damage led to immunogenic cell death (ICD) and T-cell activation. In the ER+ MCF-7 breast cancer cells, only modest DNA damage was observed, associated with apoptosis induction [35]. In breast cancer cells, combining oncolytic viruses (OVs) with G4 binders amplified cytotoxic effects, viral entry, and replication. G4 ligands and the adenovirus dl922-947 promoted senescence in cancer cells, with dl922-947 unexpectedly stabilizing G4 structures, an effect enhanced by pyridostatin (PDS). The dl922-947/PDS combination inactivated STING signaling pathways, demonstrating antitumor effects. These findings offer new insights into the mechanism of dl922-947 and suggest that pairing it with G4 ligands could help apply virotherapy to tumors that are resistant to OV-based treatments [35]. The disruption of BRCA2 alters the dynamic stability of G-quadruplex structures, leading to phase separation, where cellular components form liquid-like aggregates. These altered G-quadruplexes trigger break-induced replication, a repair mechanism that maintains telomere integrity. This process is essential for chromosome stability, and its disruption can contribute to genomic instability, often linked to cancer progression [36]. An anticancer approach targeting telomeres involves inhibiting telomerase by stabilizing the G-quadruplex structure at telomeric ends. Telomeres are typically protected by proteins like POT1 and TPP1, but when these proteins malfunction, telomeres lose their protection, leading to DNA damage and halting cell division. Small molecules can interfere with these proteins, triggering cellular responses. Transforming single-stranded telomeric DNA into a G-quadruplex structure inhibits telomerase activity. Ligands, such as telomestatin, RHPS4, BRACO-19, and TMPyP4, are effective in stabilizing the G-quadruplex in cancer cells. Importantly, TMPyP4 has also been shown to inhibit *hTERT* expression [37].

The in vitro cytotoxicity of the complexes that target human telomeric G-quadruplex motifs was assessed against several selected cancer cell lines, and the results indicated that both chiral L-/D-valine-(1,10-phen)-Cu(II) complexes exhibited significant cytotoxic effects with notably low IC50 values ranging from 1 to 3 μM. This underscores the potential of these novel copper(II) complexes as effective anticancer agents. The cytotoxic activity of the chiral L-/D-valine-(1,10-phen)-Cu(II) complexes was tested on several important human cancer cell lines, including breast cancer (MCF-7) [38].

Cytosine-rich DNA sequences have the ability to form a highly organized structure called the i-motif in slightly acidic environments. The stability of the i-motif structure provides an effective approach to inhibit the telomerase activity in cancer cells. The electrochemical biosensor was developed by modifying a carbon paste electrode with SiO_2_ nanoparticles to explore drugs capable of stabilizing this structure. Tamoxifen (Tam), an anti-estrogen hormonal drug used in breast cancer therapy, was selected as a model ligand, and its interaction with the i-motif structure was analyzed. The interaction between i-motif DNA and Tam was investigated in PBS buffer and [Fe(CN)_6_]^3−^ using cyclic voltammetry and square wave voltammetry techniques. An oxidation peak of Tam, resulting from its interaction with i-motif DNA, was detected after immobilizing the i-motif on the electrode surface. The formation of the i-motif was studied using circular dichroism spectroscopy, and the findings confirmed that this structure forms reliably at a pH of approximately 4.5 but loses stability when exposed to more physiological conditions [39].

Single-walled carbon nanotubes (SWNTs) can suppress telomerase activity by stabilizing the i-motif structure. The sustained presence of the i-motif, along with the associated G-quadruplex, ultimately results in telomere uncapping and the removal of telomere-binding proteins. This telomere dysfunction activates the DNA damage response, leading to the increased expression of p16 and p21 proteins. SWNTs demonstrated the ability to inhibit telomerase activity and disrupt telomere function in cancer cells [40].

The *hTERT* promoter exhibits a complex dynamic equilibrium involving duplex, G-quadruplex, and i-motif structures, which continuously interconvert. Cytosine residues are protected from deamination within duplex and i-motif conformations due to reduced solvent accessibility. However, during structural transitions, these residues become more susceptible to deamination than in single-stranded DNA. While i-motif formation itself does not significantly increase deamination rates compared to single-stranded DNA, the persistent structural fluctuations result in an overall ~100-fold increase in cytosine deamination susceptibility compared to duplex DNA. The dynamic structural landscape of the *hTERT* promoter, particularly the involvement of i-motifs, plays a crucial role in regulating telomerase expression and may contribute to tumorigenesis through increased mutation rates and altered gene expression [41]. Although telomerase expression and activity in cancer cells are secondary phenomena, it constitutes a limiting step in tumorigenesis [42], playing a pivotal role in cancer stemness and metastasis [43].

### 2.3. hTERT Splice Variants

Breast cancer progression and resistance to therapy are significant clinical challenges. These issues often are the result of complex molecular mechanisms that involve hTERT. The activation of telomerase, mainly through the expression of the *hTERT* gene, is a key factor that enables cancer cells to undergo unlimited proliferation. Strong increases in telomerase activity were observed in over 85% of solid tumors [44].

In breast cancer, the formation of functional telomerase depends not only on *hTERT* transcription but also on alternative splicing. The *hTERT* gene undergoes alternative splicing, resulting in multiple mRNA variants [45]. Six alternative splicing sites have been identified in hTERT mRNA—four intersections and two deletions. Among them, only the full-length transcription (α+β+) produces an active telomerase enzyme, whereas shortened variants (e.g., with α or β deletions) are either inactive or act as dominant-negative inhibitors [46]. The β splice variant acts as a negative regulator of the telomerase RNA component (TERC, also known as human telomerase RNA-hTR) as demonstrated in studies on melanoma [47].

Research performed on MCF-7 cells showed that *hTERT* expression is hormonally regulated. Estrogen can activate its transcription via the estrogen receptor (ER), while selective estrogen receptor modulators, such as tamoxifen, can antagonize these effects. 17β-estradiol has been shown to increase the expression of the α+β+ variant, whereas tamoxifen and ICI 182,780 (estrogen antagonist) reduce its expression [47].

Studies have shown the mechanisms of resistance to treatment based on a splice variant of *hTERT* in breast cancer. It was found that the most common splice variants termed α+β− or β-deletions are highly expressed in cancer cells, coding a shortened protein that is missing most of the reverse transcriptase domain but keeps the known RNA-binding motifs.

The research conducted by Imke Listerman et al. shows that the β-deletion was the *hTERT* transcript with the highest level of expression in a breast cancer cell panel. This transcript variant was associated with polyribosomes in the examined cells. The β-deletion protein binds to TERC and functions as a dominant-negative inhibitor of the endogenous telomerase. Co-overexpression of TERC restores this inhibition, indicating competition for RNA binding.

**Figure 1:**
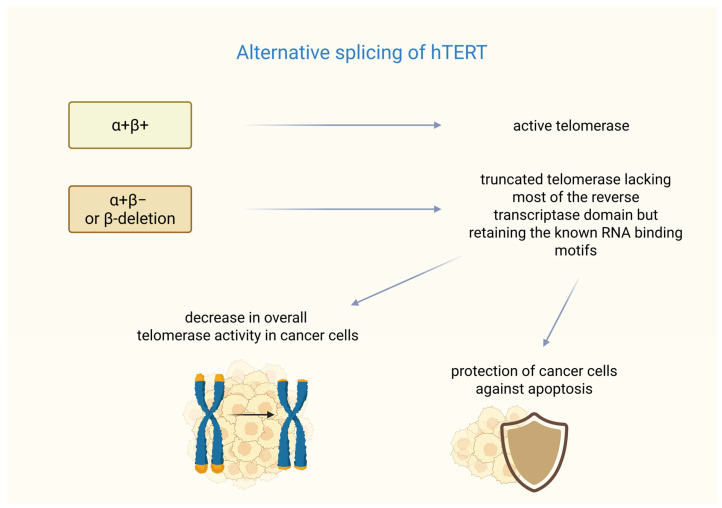
Splicing variants of *hTERT*. α+β+ (full-length transcription *hTERT*) ensures the formation of fully functional telomerase. In turn, the α+β− or β-deletion variant is responsible for the formation of telomerase lacking a significant part of its hTERT subunit. However, this hTERT variant can cause a dual effect on cancer cells—it can cause telomere shortening, but it may also protect the cells from cisplatin-induced apoptosis. Created in BioRender. Rubis, B. https://BioRender.com/izccqwt (accessed on 5 January 2026).

Despite lacking reverse transcriptase activity, β-deletion, like full-length hTERT, protects breast cancer cells from cisplatin-induced apoptosis. Its mitochondrial localization suggests that it may play a role in regulating apoptotic pathways. The alternative splicing of *hTERT* into β-deletion reduces the amount of catalytically active telomerase (RT+). Additionally, the translated β-deletion protein further reduces telomerase function, correlating with reduced telomerase activity in luminal breast cancer. The high expression of this catalytically inactive variant may help tumor cells resist apoptosis while maintaining sufficient telomere stability. This suggests potential therapeutic strategies that target β-deletion splicing to sensitize cancer cells to treatment. These findings may indicate that a major *hTERT* splice variant can give cancer cells a growth advantage that is not reliant on telomere maintenance [48]. A summary of the impact of alternative splicing of *hTERT* on telomerase function is presented in Figure 1. Unfortunately, in recent years, no clinical studies or large-scale analyses have been conducted that directly investigate the β-deletion splice variant in breast tumor tissue samples, indicating that this remains a poorly explored area, particularly with respect to in vivo studies in patients.

## 3. Epigenetic and Genomic Alterations Affecting Telomerase Regulation

Telomerase expression is governed by epigenetic regulation mediated by DNA methylation, histone modifications, and non-coding RNAs, as well as genetic alterations such as chromosomal rearrangements that are associated with aberrant *TERT* activation in cancer.

### 3.1. Epigenetic Regulation of hTERT Expression

Epigenetic modifications, such as DNA methylation, histone modifications, and non-coding RNAs, are significant in breast cancer development and progression [49]. DNA methylation and histone modifications regulate *hTERT* expression at the transcriptional level, while non-coding RNAs, such as microRNA, influence it post-transcriptionally [50]. Unlike genetic mutations, epigenetic aberrations are potentially reversible, making them promising therapeutic targets in cancer treatment [51].

#### 3.1.1. DNA Methylation

DNA methylation is essential for regulating key biological processes, including chromatin remodeling, genomic imprinting, X-chromosome inactivation, post-translational modifications, transcriptional regulation, and post-transcriptional control [52]. Cancer is characterized by abnormal DNA methylation patterns [53]. The hypermethylation of promoter CpGs can lead to the inactivation of tumor suppressor genes, while the hypomethylation of oncogenes promotes their aberrant activation [49].

Promoter methylation is a key regulator of *hTERT* expression in cancer cells. The *hTERT* core promoter lies within a 4 kb CpG island (~70% GC content), extending from −1800 to +2200 bp relative to the transcription start site (TSS) [54]. In various cancer cell lines, including breast cancer, hypermethylation occurs approximately 600 bp upstream of the *hTERT* promoter, while the region near the TSS remains unmethylated. The presence of active chromatin marks (acetyl-H3K9, dimethyl-H3K4) in the unmethylated TSS region suggests its essential role in *hTERT* induction, whereas hypermethylation correlates with inactive chromatin states (trimethyl-H3K9, trimethyl-H3K27) [55]. The *hTERT* hypermethylated oncological region (THOR) plays a crucial role in *hTERT* regulation. While an unmethylated THOR suppresses *hTERT* promoter activity, its hypermethylation promotes its activation. Up to 75% of breast cancer cases exhibit THOR hypermethylation [56]. Research by Teisha J. Rowland et al. confirmed this pattern in 23 different types of cancer tissues, reinforcing its significance [57]. Recent findings indicate that THOR hypermethylation in breast cancer tissues may serve as a potential biomarker and therapeutic target for early diagnosis [58]. Further research is required to explore *hTERT* DNA methylation dynamics across different breast cancer subtypes and progression stages.

#### 3.1.2. Hypermethylation as a Biomarker

Higher hypermethylation was observed in breast tumor cells, especially in four CpG islands, compared to normal breast tissue. These particular CpG islands exhibited more than 50% methylation in approximately three-quarters of the 77 breast cancer samples. These findings show that DNA hypermethylation of the *hTERT* promoter region at −600 bp may be a diagnostic marker for breast cancer [59].

Recent studies have shown that the methylation of the *hTERT* gene can serve as a biomarker for predicting the effectiveness of all-trans retinoic acid (ATRA) treatment in breast cancer. This therapy consists of repressing *hTERT* expression. Evidence suggests that specific methylation patterns in the *hTERT* gene, particularly at CpG8 in the −5 kb upstream region, could be valuable predictive markers for the therapeutic response to ATRA.

These observations emphasize the importance of considering DNA methylation profiles in personalized therapeutic strategies, especially when we focus on how breast cancer subtypes have variable responses to ATRA. In ER+ cancers, higher methylation in *hTERT* region II (from the position −5500 to −4900 bp relative to the transcriptional site star) supports telomerase expression and activity, while in triple-negative breast cancer (TNBC), lower methylation indicates greater resistance to ATRA. The methylation level of *hTERT* influences its suppression and treatment response. CpG region methylation serves as a biomarker indicating the potential success of treatment in specific breast cancer cell lines: MCF7 (ER+), T47D (ER+), ZR75.1 (ER+), and SKBR3 (HER2+) [60]. Hypermethylation of the *hTERT* gene may be considered as a tool to monitor therapy but also as a predictive marker of treatment efficacy and response, for example, in the case of ATRA therapy.

Despite the potential of THOR and *hTERT* hypermethylation mechanisms as biomarkers, the translation of DNA methylation findings into in vivo models remains challenging. Factors such as interindividual biological heterogeneity, tumor subtype diversity, and methodological variability can affect biomarker reproducibility and accuracy [61]. For example, detection of *hTERT* methylation in circulating DNA is inconsistent among breast cancer patients, highlighting the translational gap between cell line- and tissue-based studies and clinically applicable liquid biopsies [62]. Moreover, different methylation profiles indicate that biomarker reliability may vary across breast cancer molecular subtypes, complicating their universal application [63]. Technical limitations, such as low circulating tumor DNA (ctDNA) levels and background noise, further constrain the sensitivity of methylation-based diagnostic approaches [64].

#### 3.1.3. Histone Modifications

Histone modifications, including acetylation/deacetylation and methylation/demethylation, play a crucial role in chromatin accessibility and gene expression. In breast cancer, aberrant histone modifications, often coupled with DNA hypermethylation, contribute to the epigenetic silencing of tumor suppressor genes and genomic instability [65]. Active histone marks, such as dimethyl-H3K4, trimethyl-H3K4, and acetyl-H3, promote gene expression, whereas inactive marks (trimethyl-H3K9 and trimethyl-H3K27) are associated with repression [55,66]. Recent studies highlight the impact of histone modifications on *hTERT* expression in breast cancer cells. For instance, genistein has been shown to increase trimethyl-H3K9 (inactive mark) and decrease dimethyl-H3K4 (active mark), leading to *hTERT* suppression. However, clinical studies evaluating the epigenetic effects of genistein (e.g., trimethyl-H3K9 or dimethyl-H3K4) directly in patients with active breast cancer are lacking [67]. Similarly, centchroman (used as an oral contraceptive pill) downregulates histone deacetylase (HDAC) expression, which enhances active acetylation marks (acetyl-H3, acetyl-H4) and reduces repressive modifications, ultimately decreasing *hTERT* expression [68]. These studies suggest a complex interplay between DNA methylation and histone modifications in *hTERT* regulation. Further research is needed to fully understand this interaction, especially at the *hTERT* promoter.

#### 3.1.4. Non-Coding RNAs

The role of non-coding RNAs (ncRNAs) in breast cancer is still being explored, but their significance is becoming increasingly evident. NcRNAs, including microRNAs (miRNAs), are frequently dysregulated in cancer, contributing to cancer-specific epigenetic signatures. These molecules regulate factors such as DNA methyltransferases (DNMTs) and histone deacetylases (HDACs), influencing processes such as proliferation, differentiation, invasion, and metastasis [49]. MicroRNAs are small RNA molecules (19 to 24 nucleotides) [69] that bind to the 3′ untranslated region (UTR) of mRNA, leading to its silencing and a reduction in gene expression. MicroRNAs that target *hTERT* mRNA have been shown to directly decrease *hTERT* expression, while others may indirectly affect hTERT levels. As a result, microRNAs can function as tumor suppressors or promoters, significantly influencing *hTERT* expression and cellular functions. For example, reduced levels of miR-296-5p and miR-512-5p have been observed in breast cancer. The silencing leads to increased *hTERT* expression in basal-type breast cancer cells, which is related to worse prognosis [70].

#### 3.1.5. miRNAs as Biomarkers

The miRNA levels can be used as a biomarker of epigenetic modification. As it was demonstrated above, reduced levels of miR-296-5p and miR-512-5p reflect epigenetic silencing of genes corresponding to these miRNAs, which leads to overexpression of *hTERT*. Determining the levels of specific miRNAs can predict treatment response, prognosis, and may indicate potential targets for genetic therapeutic interventions [70].

Increased methylation occurs in regions coding for miRNA-hosting genes and CpG islands with a regulatory role in miR-296-5p and miR-512-5p expression. Previous studies have shown that glioblastoma stem cells treated with the DNMT inhibitor, 5-aza-2′-deoxycytidine, reduced DNA methylation levels of CpG-rich regions of interest and increased levels of miR-296-5p and miR-512-5p. Re-expression of miR-512-5p enhanced cisplatin-induced apoptosis and inhibited cell migration. Additionally, miR-512-5p reduced cell proliferation. It should be emphasized, however, that no direct in vivo studies have been conducted in patients with breast cancer, and the available evidence is primarily derived from in vitro experiments and cell line expression analyses. Therefore, it remains unclear whether the observed regulatory mechanisms of miR-296-5p and miR-512-5p, as well as the effects of DNA demethylation, are recapitulated in the clinical setting, across different breast cancer subtypes, and within the complex tumor microenvironment [71].

Treatment with 5-azacytidine and trochostatin A (5′aza + TSA) reduces CpG island methylation, which leads to the re-expression of previously silenced microRNAs. This change in gene regulation triggers a series of cellular effects, including the induction of apoptosis, inhibition of cell growth, and suppression of cell migration. Together, these changes may help reduce tumor malignancy. The biological outcomes are due to the inhibition of TEAD4, a transcription factor, through the action of miR-512-5p [72].

Similar conclusions came from studies on breast cancer cells. Researchers found that miR-296-5p, miR-512-5p, and miR-1207-5p are significantly downregulated in breast cancer compared to normal tissue. These miRNAs reduce *hTERT* 3′ untranslated region (3′UTR) reporter activity by at least 50%. 3′ UTRs regulate mRNA processes. MiR-296-5p has shown tumor-suppressive properties in many cancers, including breast, prostate, non-small-cell lung cancer, and glioblastoma. MiR-512-5p activates apoptotic pathways in lung and gastric cancer and targets *hTERT* in head and neck squamous cell carcinoma. The Lowe levels of miR-2965p and miR-512-5p result from the epigenetic silencing of their genes. Reversing this silencing also raises miRNA levels, lowers *hTERT* expression, and increases apoptosis, suggesting a potential role in basal-like breast cancer (BLBC) therapy [70]. The silencing of miR-296-5p and miR512-5p allows for *hTERT* overexpression, which contributes to BLBC progression and poor patient outcomes.

MiRNAs can be used as biomarkers to show which genes are silenced. This method could be used to monitor how effective treatments are in reversing epigenetic silencing.

The epigenetic regulation of *hTERT* through DNA methylation and miRNAs offers valuable biomarkers of breast cancer progression and response to treatment. Although not suitable for early diagnosis, these markers support more personalized and targeted therapeutic strategies. Markers, such as miR-296-5p, miR-512-5p, and hypermethylation in the *hTERT* gene, are worthy of consideration and further research.

**Figure 2:**
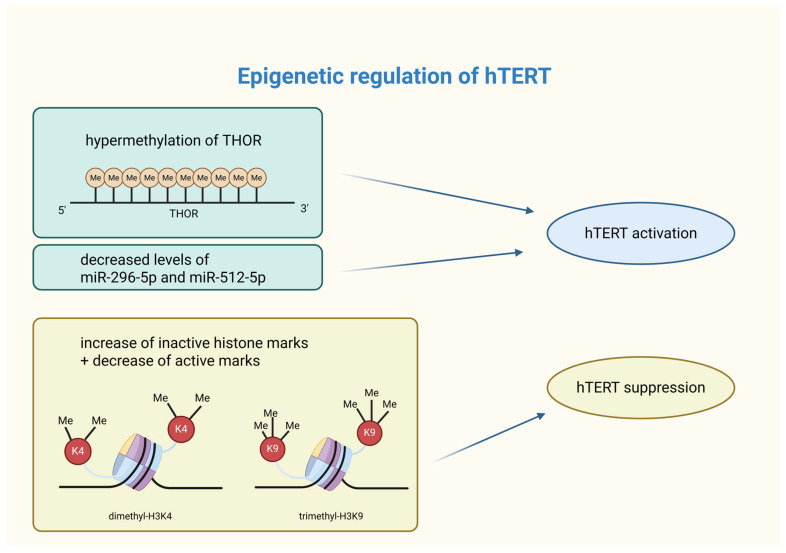
Summary of epigenetic modifications affecting hTERT activity. Epigenetic modifications are capable of shaping hTERT activity. Its activation in cancer cells can occur through THOR hypermethylation or silencing of specific miRNAs. On the other hand, *hTERT* suppression is caused by an increase in inactive histone marks (such as dimethyl-H3K4) and a decrease in active histone marks (i.e., trimethyl-H3K9). hTERT—human telomerase reverse transcriptase; THOR—hTERT tumor hypermethylated oncological region; Me—methylation; miR—microRNA; H3K4—lysine 4 at histone 3; K4—lysine 4; H3K9—lysine 9 at histone 3; K9—lysine 9. Created in BioRender. Rubis, B. https://BioRender.com/unvj7ma (accessed on 5 January 2026).

A graphical summarization of the influence of epigenetic processes on hTERT activity is shown in Figure 2.

### 3.2. Chromosomal Rearrangements Affecting Telomerase Expression

The telomerase complex consists of the telomerase reverse transcriptase (TERT) protein and the telomerase RNA (TR) [73]. The *TERT* gene has been identified and mapped, using the fluorescence in situ hybridization (FISH) method, to the location of chromosome 5p15.33 [74].

In contrast to the majority of differentiated human cells, where the *TERT* gene is repressed, elevated expression of telomerase has been observed in up to 90% of human malignancies and most immortalized human cell lines. These kinds of observations imply a connection between increased telomerase activity and carcinogenesis. Several studies suggest that chromosomal aberrations, such as translocations, may, in some cases, be linked to abnormal activation of the *TERT* gene [75,76,77].

Yuan et al. present the results of another analysis that examined, among other things, structural alterations and expression of *TERT* in 31 cancer types derived from The Cancer Genome Atlas cohort of patients. Translocations to other chromosomes were observed. One of the malignancies taken under consideration was breast invasive carcinoma (BRCA), in which the result of the examination of structural variants and *TERT* gene amplification was approximately 4% [75].

A study surrounding the correlation between chromosomal aberrations targeting the *TERT* gene and the occurrence of B-cell neoplasms shows possible evidence of *TERT* deregulation caused by translocations to *IGH* and non-*IG* loci. Nagel et al. observed the same translocation transpiring in three cases of chronic lymphocytic leukemia and one case of precursor B-cell acute lymphoblastic leukemia, all of which affected the 5p15.33 region containing the *TERT* gene. In those four cases, *TERT* was translocated to a derivative chromosome 14, evidenced by FISH mapping. One instance showed that the *IGH-TERT* translocation was the only mutation occurring, which may imply a key role of *TERT* abnormalities in lymphomagenesis [38].

One analysis of 34 patients with acral lentiginous melanoma (ALM) showed somatic *TERT* translocations in a total of 14 (41%) cases. *TERT* breakpoints identified by long-insert whole-genome (LIWG) sequencing were displayed in four patients. An extensive rearrangement in chromosome 5, which contained a translocation impacting exon 11 and an inversion in intron 11, was observed in one of the cases. The intra-exon translocation taking place was supported by RNA reads. The mutations were additionally accompanied by increased *TERT* expression in the tumor [78].

Another research concerning the identification of the mutational landscape of hepatocellular carcinoma revealed two examples of *TERT* translocations, presumably leading to activation of the gene’s expression. In one patient, the intrachromosomal translocation of *TERT* was caused by massive genomic rearrangements of chromosome 5. The second patient’s aberration involved the promoter region of *TERT*, which was translocated to chromosome 9. In the first case, translocation exhibited *TERT* expression in pathological tissue, as opposed to a non-tumor sample (there was no available data for the other case) [79].

## 4. Non-Canonical Roles of Telomerase in Breast Cancer Cells

Telomerase complex elements are responsible not only for telomerase activity and telomere restoration. As demonstrated, they may also contribute to some other telomere-independent mechanisms that affect cell metabolism, irrelevant to telomerase activity.

### 4.1. Cellular Translocation

Studies performed using immunohistochemistry techniques demonstrated that hTERT, in addition to its localization in the nucleus, is also expressed in the cytoplasm [80].

Research conducted by Yuji Uno et al. suggests that hTERT, which is typically localized in the nucleus, can dynamically translocate to the cytoplasm, particularly in HER2+ breast cancer following primary systemic therapy (PST). This cytoplasmic redistribution of hTERT has been observed more frequently after PST in HER2+ tumors, implying a potential role in mediating resistance to therapy [81]. These findings support the hypothesis that the cytoplasmic localization of hTERT may contribute to treatment resistance mechanisms. Research performed by Hannen et al. also confirmed this thesis [43].

Another research group demonstrated that in malignant cells put under exogenous stress, hTERT is excluded from the nucleus and translocated to the mitochondria, which distinguishes them from non-cancerous cells, where that exclusion usually did not take place or was minimal [82,83,84]. This occurrence has been linked to protection against DNA damage as well as apoptosis resistance in cancerous cells [85].

A study set to document the kinetic process of the cellular hTERT translocation after introducing H_2_O_2_ treatment in three different cell lines, one of which is a human breast cancer line, MCF-7. The shuttling of telomerase started roughly 45 min after administering the treatment and reached an exclusion level of 50–60% the following day, in the mentioned cell line. This analysis revealed that, in all three lines, cells that excluded hTERT had significantly lower levels of damaged DNA in comparison to cells with regular levels of nuclear telomerase present. Singhapol et al. believe that mitochondrial translocations of hTERT are a protective mechanism against exogenous stress, such as anti-cancer therapeutic treatment, which can lead to increased resistance in cancer cells [82].

Using immunofluorescence staining and confocal laser microscopy, Ling et al. were able to demonstrate the shuttling of hTERT into the mitochondria of multidrug-resistant (MDR) cell lines prepared from hepatocellular carcinoma cells. The results indicate a possible correlation between increasing resistance indices of these MDR cell lines and increased mitochondrial expression of *hTERT*. Telomerase, translocated from the nucleus and having a role in protecting mitochondria, would explain why the amplification frequency of mtDNA, and consequently mtDNA damage, in parental cells was higher than in drug-resistant cells [84].

Taking into account the protective effect of hTERT mitochondrial translocations, Mazloumi et al. conducted research focused on triple-negative breast cancer (TNBC) cell lines. These lines are characterized by high telomerase activity and mitochondrial biogenesis, which contribute to the exceptionally invasive and therapy-resistant nature of this cancer. Treating cells with a telomerase inhibitor (BIBR1532) and a mitochondria inhibitor (tigecycline) resulted in induced apoptosis without significant changes in telomere lengths, which is why, according to this study, the combined inhibition of telomerase and mitochondria respiration could be utilized in future breast cancer treatment [85].

hTERT contributes not only to tumor development, but also to therapy resistance. Drug resistance mechanisms in breast cancer involving hTERT are complex and not fully understood. It is important to recognize their significance and complexity to improve treatment strategies. While hTERT is mainly known for its role in maintaining telomeres, dynamic changes in subcellular localization show additional functions. Overall, hTERT drives breast cancer progression, providing promising targets to overcome drug resistance.

### 4.2. Role of Telomerase in Mitochondria

Telomerase is a crucial enzyme responsible for maintaining telomere length, but it has also been proven to be present in mitochondria, where it can influence other cellular processes, including the response to cancer treatment.

**Figure 3:**
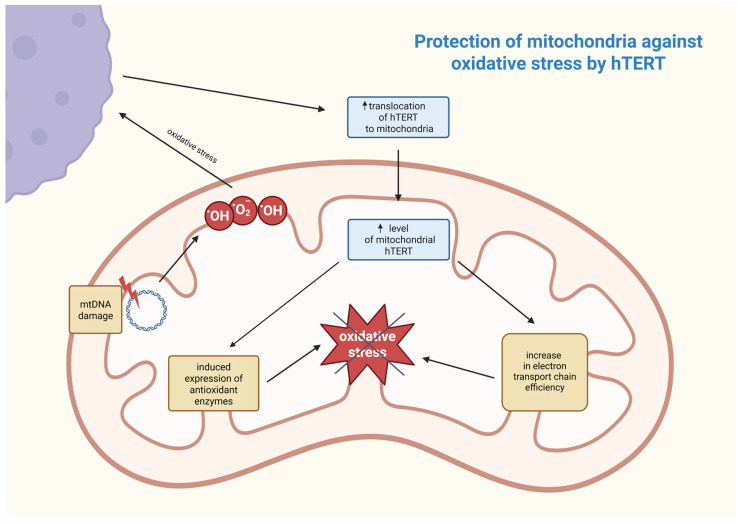
Postulated mechanism of mitochondrial defense against oxidative stress involving hTERT. The increase in ROS (e.g., hydroxyl and superoxide anion radicals) as a result of mtDNA damage contributes to an increase in hTERT translocation from outside the mitochondrion and, as a consequence, a reduction in ROS responsible for oxidative stress occurs in a feedback loop. hTERT—human telomerase reverse transcriptase; mtDNA—mitochondrial DNA; ^•^OH—hydroxyl radical; ^•^O_2_^−^—superoxide anion radical. Created in BioRender. Rubis, B. https://BioRender.com/9a2rbxh (accessed on 5 January 2026).

First, understanding the sub-mitochondrial localization of hTERT is crucial for determining its molecular function. Research work was conducted wherein it was discovered that hTERT was mostly located in the mitochondrial matrix. This was established by digesting mitochondria with proteinase K and subsequently examining for hTERT by using Western blots [86]. Mitochondrial DNA (mtDNA) is also an important component of the mitochondrial matrix, and although direct proof is still lacking, it is suspected that hTERT may be involved in mtDNA metabolism. This speculation may also be reinforced by recent studies demonstrating that hTERT binds to mtDNA [86,87]. The first known function of telomerase in mitochondria is protection against oxidative stress by reducing levels of reactive oxygen species (ROS). Under oxidative stress, mitochondrial hTERT levels rise, and this process is regulated by post-translational modifications. This suggests its adaptive protective function that is controlled by Src kinase and can be reversed by dephosphorylation of hTERT by the tyrosine phosphatase Shp-2 [83,87]. Damage to mtDNA leads to increased ROS generation, and hTERT enhances the efficiency of the electron transport chain, reducing electron leakage and oxidative stress. It also induces the expression of antioxidant enzymes, such as manganese superoxide dismutase (MnSOD), catalase, and glutathione peroxidase, which neutralize ROS [86]. The association between these processes is shown in Figure 3.

As previously mentioned, hTERT binds to mtDNA, significantly raising mtDNA stability. It occurs through participating in mtDNA repair mechanisms, supporting its integrity, and reducing mutation frequency. However, it is only possible with the protein being present in the organelle [83]. Additionally, hTERT can contribute to the transcription of mitochondrial genes by binding to mtDNA, strengthening genome stability, upon which mtDNA transcription heavily depends. Simultaneously, hTERT reduces levels of ROS, ameliorates electron transport chain efficiency, and enhances antioxidant defenses, resulting in mtDNA remaining more transcriptionally competent. Through these mechanisms, hTERT plays an essential role in maintaining mitochondrial homeostasis, which may be significant in the context of cancer, neurodegenerative diseases, or infertility treatment [88]. hTERT contributes to drug resistance in cancer cells by protecting mitochondrial DNA and reducing oxidative stress, thereby promoting cell survival. The increased translocation of mitochondrial hTERT in hepatocellular carcinoma cells increases their resistance to chemotherapeutic agents by inhibiting mitochondrial apoptosis, as evidenced by increased mitochondrial membrane potential, elevated mtDNA copy number, and reduced caspase-3 activation. Consequently, cancer cells with high mitochondrial hTERT expression exhibit reduced sensitivity to pharmacological treatments, posing a great challenge for effective therapy in cancers, such as hepatocellular carcinoma [89]. In contrast, after carbon-ion irradiation, telomerase activity in MCF-7 breast cancer cells was suppressed via *hTERT* downregulation, which was confirmed by RT PCR and Western blot tests. Simultaneously, it was established that the activity of other telomerase subunits—TERC and TP-1—was not significantly changed, nor was the telomere length within 10 days post carbon-ion irradiation. Since telomerase activity is found in about 90% of cancer cells and it plays a key role in helping these cells maintain their immortalization, *hTERT* suppression meaningly increases radiosensitivity, which could be further increased by telomerase inhibitor MST-312 [88]. Normally, about 10 to 20% of the total cellular amount of hTERT is mitochondrial, while under oxidative stress, up to 80% of the protein could be found within mitochondria. This accumulation of hTERT protects mitochondria under oxidative stress and could be indicated by the observed rise in mitochondrial membrane potential, decreased cell peroxide levels, and mitochondrial superoxide production. Carbon-ion irradiation disrupts this protective role of hTERT in mitochondria, leading to mitochondrial dysfunction and increased levels of ROS. This leads to cellular senescence, which is the primary mechanism of MCF-7 cell inactivation after irradiation [88]. As shown earlier, apart from telomerase’s canonical role in telomere length maintenance, telomerase’s catalytic subunit hTERT also localizes in the mitochondrial matrix, where it plays a very crucial non-canonical role in enhancing mtDNA stability and protecting mitochondria from oxidative stress by improving electron transport chain efficiency and inducing antioxidant enzymes. By these processes, hTERT supports mitochondria homeostasis and, through that, reinforces cancer cells’ survival as well as their resistance to drugs. This suggests that suppressing telomerase activity in MCF-7 cancer cells by *hTERT* downregulation via carbon-ion irradiation might be used in developing cancer treatments.

## 5. Translational Potential of Telomerase-/Telomere-Targeting Approach

Telomerase (and telomere) structure and roles are complex. hTERT’s canonical role (telomere restoration after each cell division) and non-canonical roles (a wide spectrum is postulated, including gene expression regulation, anti-apoptotic potential, DNA repair, and metabolism control) work together, creating complex signaling networks. Eventually, all these processes enhance cancer cell survival and proliferation, leading to therapy resistance by stabilizing telomeres, boosting antioxidant defenses, activating pro-survival pathways like NF-κB, and interfering with cell death signals [90,91,92].

All these aspects make telomerase-/telomere-targeting either function alone insufficient, requiring dual strategies for effective cancer treatment. Even if telomerase function is compromised, another telomere restoration mechanism can be reinforced, i.e., Alternative Lengthening of Telomeres (ALT), which appears in up to over 60% of all cancers, depending on the cancer type [93]. Additionally, this mechanism can be observed in telomerase-positive cells, and the transition between these two is not fully understood [94]. Furthermore, the heterogeneity of tumor cells, accompanied by the existence of ALT and telomerase activity in different cells of the same tumor, is reflected by the very diverse telomere length in those cells [95,96,97].

Consequently, it also makes the selection of a cancer strategy more difficult. In turn, the non-canonical roles of telomerase in cancer refer to the ability of hTERT to bind to promoters of genes that are involved in growth (like c-Myc) and metastasis (like EMT markers), independent of telomeres. Telomerase is associated with cell survival by blocking cell death signals, increasing resistance to chemotherapy. It can also boost DNA damage repair pathways, helping cancer cells recover from therapy-induced damage. Additionally, telomerase regulates mitochondrial function, increasing efficiency and antioxidant capacity (e.g., via glutathione, ROS) to combat oxidative stress from treatment [14,91,92,98,99,100].

Most importantly, the canonical and non-canonical functions of telomerase can collaborate. Due to non-canonical functions, some signaling pathways are activated (like NF-κB, Src) that, in turn, can upregulate *hTERT* expression and activity, creating positive feedback loops that enhance cancer progression and drug resistance [101].

Even if telomeres are targeted (which is supposed to limit the access of telomerase to telomeres), non-canonical functions of hTERT (like antioxidant support) still allow cells to survive and repair damage, while inhibiting the non-canonical roles might not stop telomere maintenance. Thus, a postulated efficient therapeutic approach should involve and combine synergistically addressing both roles. A critical question remains whether such an approach is clinically actionable at all; it seems that the utilization of compounds that show diverse effects, like TMPyP4, which is known to inhibit telomerase (via G4 intercalation) but also to downregulate *hTERT* promoter, is an interesting option. Additionally, this compound is capable of revealing photodynamic therapy benefits that trigger reactive oxygen species (ROS) that damage tumor cells directly. Regarding the fact that this porphyrin can also decrease *hTERT* expression, it seems that it may significantly contribute to cancer elimination. However, so far, the telomere-targeted drugs approved in clinical trials mainly fall into two categories: chemotherapeutic and immunotherapeutic agents. Chemotherapeutics include telomerase inhibitors (e.g., Imetelstat, KML-001) and telomere dysfunction inducers (e.g., nucleoside analog 6-thio-2′-deoxyguanosine). Immunotherapeutics include telomerase-specific oncolytic adenoviruses (e.g., OBP-30141–44, KH901), hTERT peptide vaccines (e.g., GV1001), and hTERT mRNA vaccines [102]. At present, it is difficult to estimate their efficacy, but the number of studies on this subject confirms the importance of this approach.

## 6. Conclusions

Breast cancer remains a significant clinical challenge because of high incidence and mortality rates. Telomerase, especially its reverse transcriptase subunit (hTERT), is crucial for the growth and survival of cancer cells. hTERT’s primary role in keeping telomere stability is well known, but we need to better understand the different modes of its modifications and non-canonical functions, especially in the context of aging and cancer.

Its common expression in tumors and significant role in the response of cancer cells to therapy make hTERT a critical candidate in diagnostics and treatment. It seems that the phrase that refers to telomerase (“Balancing on the Edge”) accurately reflects the complex role of telomerase. When telomerase functions within physiological conditions, it supports normal cell division; however, its overexpression is commonly known to be associated with carcinogenesis. Understanding these multifaceted mechanisms behind hTERT’s role in (breast) cancer progression and therapy resistance is crucial for developing more effective therapeutic strategies.

Overall, there is a notable lack of in vivo studies addressing both the canonical and non-canonical functions of telomerase in breast cancer within the aspects discussed in this review. It mainly results from differences between in vitro and in vivo models involved (Table 2).

The directions for telomerase investigation demonstrated in this review are based on recent scientific reports that likely explain the current lack of translational effects and implementation in clinics. Even if we miss standardized criteria for advancing from preclinical to clinical trials, there is a common conviction that in vitro data can predict in vivo efficacy under appropriate pharmacokinetic and tumor-specific conditions [103]. That, in turn, highlights the need for a comprehensive systematic approach and targeted in vivo research. Systematic reviews and meta-analyses indicate that animal models can provide valuable preclinical data. Although variability in model responses requires careful study design and greater methodological transparency, these models can serve as an important intermediate platform bridging in vitro and in vivo research [104].

## Figures and Tables

**Table 1:** Summary of research models in studies on modulators of telomerase activity in breast cancer.

Group of Tested Compounds	Name of the Compound	Research Model	Source
Oligonucleotides	Imetelstat (GRN163L)	A group of 10 males and females with pathomorphologically confirmed HER2+ breast adenocarcinoma	Clinical trial NCT01265927, 2015 [18]
		HCC1569, HCC1954, SKBR3, SKBR3-R (trastuzumab resistant), and TMD-231 breast cancer cell lines	Koziel et al., 2015 [19]
		24 histologically or cytologically confirmed breast adenocarcinoma patients with measurable locally recurrent or metastatic disease	Clinical trial NCT00732056, 2015 [21]
	T-oligos	MMT mice generated by crossing MUC1 transgenic mice with MMTV-PyMT mice, expressing polyomavirus middle T oncogene and developing spontaneous mammary carcinomas	Weng et al., 2010 [22]
Synthetic compounds	Barasertib (AZD1152-HQPA)	SK-BR-3 and MCF-7 cell lines	Tara et al., 2025 [23]
Endogenous compounds	Insulin-like growth factor binding protein-3 (IGFBP-3)	MCF-7 breast cancer cell line	Kwon et al., 2023 [27]
Plant compounds	Boldine	Different cell lines, including MCF-7	Kazemi Noureini et al., 2015 [28]
	Helenalin	T47D breast cancer cell line	Barkhordari et al., 2023 [29]
	Curcumin	MCF-7 cell line	Fawzy et al., 2024 [30]
	Bisphenols A (BPA), F (BPF), and S (BPS)	MCF-7 and MDA-MB-231 cell lines	Awada et al., 2020 [31]

**Table 2:** Comparison of in vitro and in vivo studies on role of telomerase in breast cancer.

Aspect	In Vitro Studies	In Vivo Studies
Experimental control	Higher level of control over experimental conditions (precise manipulation of single variables)	Limited control due to systemic interactions
Ethical concerns	Fewer ethical considerations	The necessity of obtaining permits and a positive approval from the bioethics committees
Mechanistic insight	Good for analyzing molecular mechanisms and signaling pathways	The analysis of mechanisms may be disrupted by the influence of interacting factors
Cost and time	Relatively low-cost and rapid execution	Higher cost and longer experimental timeline
Reproducibility	Generally high reproducibility	Greater biological variability between individuals
Translational relevance	Limited ability to predict clinical outcomes	Increased translational value, despite inherent species-specific limitations
Drug response assessment	Useful for initial screening and dose–response analyses	Enables assessment of pharmacokinetics, toxicity, and systemic effects

## Data Availability

No new data were created or analyzed in this study.
